# Hooded Crows (*Corvus cornix*) May Be Aware of Their Own Body Size

**DOI:** 10.3389/fpsyg.2021.769397

**Published:** 2021-12-16

**Authors:** Ivan A. Khvatov, Anna A. Smirnova, Maria V. Samuleeva, Evgeniy V. Ershov, Svetlana D. Buinitskaya, Alexander N. Kharitonov

**Affiliations:** ^1^Moscow Institute of Psychoanalysis, Moscow, Russia; ^2^Department of Higher Nervous Activity, Faculty of Biology, Lomonosov Moscow State University, Moscow, Russia; ^3^Institute of Psychology, Russian Academy of Sciences, Moscow, Russia; ^4^Institute of Experimental Psychology, Moscow State University of Psychology and Education, Moscow, Russia

**Keywords:** self-awareness, body awareness, body size awareness, mirror self-recognition, hooded crows

## Abstract

Body-awareness is one of the manifestations of self-awareness, expressed in the ability of people and animals to represent their own body physical properties. Relatively little work has been devoted to this phenomenon in comparison with the studies of the ability of self-recognition in the mirror, and most studies have been conducted on mammals and human infants. Crows are known to be “clever” birds, so we investigated whether hooded crows (*Corvus cornix*) may be aware of their own body size. We set up an experimental design in which the crows had to pass through one of three openings to reach the bait. In the first experiment, we studied whether crows prefer a larger hole if all the three are suitable for passage, and what other predictors influence their choice. In the second experiment, we assessed the ability of the crows to select a single passable hole out of three on the first attempt, even though the area of the former was smaller than that of the other two. The results of the first experiment suggest that when choosing among three passable holes, crows prefer those holes that require less effort from them, e.g., they do not need to crouch or make other additional movements. In the second experiment, three of the five crows reliably more often chose a single passable hole on the first try, despite its smaller size. We believe that these results suggest that hooded crows may be aware of their own body size.

## Introduction

Self-awareness is the ability of an individual to distinguish its self from the environment and to separate the “self-entity” from the “other-entity.” In its rudimentary form self-awareness is the ability to become the object of one’s own attention and the capacity to ascribe properties to oneself (Gallup et al., 2002).

There are three main approaches to the research in of self-awareness in animals: (1) mirror self-recognition (MSR; e.g., Gallup and Anderson, 2020); (2) the ability of animals to distinguish their own odor (“olfactory mirror,” Horowitz, 2017; Gatti et al., 2020), and (3) the ability of animals to represent their own body’s physical properties (body-awareness paradigms; Dale and Plotnik, 2017; Lenkei et al., 2020, 2021). Self-awareness is closely related to the ability to infer mental states, such as desires and beliefs, in others (“theory of mind”; Gallup, 1982; Gallup et al., 2002; Krupenye and Call, 2019). For an objective assessment of the level of development of self-awareness of an individual or species, it is necessary to analyze the results obtained using as many approaches as possible.

The fact that a subject has an idea of the physical properties of its body may be evidenced by the ability to spontaneously (without special training) solve problems for which these properties are needed. Currently, two variants of such tasks are used. One is used to assess the ability of children, elephants and dogs to operate with the idea that their body has weight and to understand that body weight can be an obstacle to the performance of an experimental task (Brownell et al., 2007; Dale and Plotnik, 2017; Lenkei et al., 2021).

The second approach allows the researcher to assess the subjects’ knowledge of its own size, that is whether they can correlate it with the size and shape of the hole through which they are supposed to pass (Brownell et al., 2007; Lenkei et al., 2020). The advantage of this approach is its potential applicability to a wide range of species. However, when using it, the same problem arises as when using other methods aimed at studying the thinking of animals, i.e., how to distinguish operating with representations from fast learning to solve an experimental problem. Further, we consider how this problem was addressed in three major studies conducted to date (Brownell et al., 2007; Dale and Plotnik, 2017; Lenkei et al., 2021).

In the work with children (Brownell et al., 2007), a child standing on a blanket was presented with the task to push a stroller to which the blanket was attached. In order to fulfill the task, the child had to get off the blanket (an attempt to push the stroller without leaving the blanket was regarded as an erroneous action). In another test, a child sat on a mat and listened to a short story. When the story ended, the experimenter asked the child to handover the mat it was sitting on. The attempts to pull out the mat from under its body without moving first were considered an erroneous action. Children aged 18 months coped with both variants of the tasks only after one, and more often several, erroneous actions. At the age of 22–26 months, the number of erroneous actions significantly decreased, and some children solved these problems from the first try. These results suggest that the idea of the properties of one’s body (namely, that it has weight) is just beginning to form during the second year of life. Similar findings were reported by Moore et al. (2007) from a study where children had to get off a rug to move a cart on the floor. The authors note that significant shifts in the development of perception of their own body occurs in children around the age of 18 months.

In the work with elephants (Dale and Plotnik, 2017), the animals were preliminarily trained to pick up a stick and give it to the experimenter. The experiment involved 12 elephants. 48 background trials and 12 test trials were carried out with each animal. At the beginning of each trial, the elephant was brought onto a mat. In test trials, a stick was tied to the mat. The experimenter stood at such a distance from the mat that it was only possible to pass the stick over to him by getting off the mat. Background conditions, in which the stick was not tied to the mat, made it possible to find out whether the elephants left the mat only when it was necessary to solve the problem. Two types of background trials were used that differed only in that in one of them the experimenter pulled on a rope tied to the mat creating a tension on the fabric under the elephant’s feet, whereas in the other condition no rope was used. There were 24 trials of each type, that showed no significant difference between the conditions. Comparison of the results of all 48 trials in background conditions with 12 test trials showed that in test trials the elephants significantly more often left the mat only when it was necessary. Four elephants did not make a single error in 12 test trials, four more animals made only one mistake. These results indicate that the correct action in test trials was not formed as a result of learning. Thus, elephants have been shown to exhibit evidence for possessing at least two components of self-awareness: the understanding of the physical properties of their own body (that their own body has weight: Dale and Plotnik, 2017) and of mirror self-recognition (Plotnik et al., 2006). In addition, numerous observations indicate the development of empathy in them, which is considered an integral part of the theory of mind (Bates et al., 2008).

A similar technique was used to assess body awareness in 54 dogs (Lenkei et al., 2021). In this study, the dogs were presented with the same test conditions as the elephants (Dale and Plotnik, 2017) and in addition implemented a third type of control condition in which the stick was tied to a hook fixed in the ground next to the mat, which did not create “foot discomfort” as when attached to the mat. To reduce the effect of training, each dog was presented with 4 sessions in each condition (4 test and 12 control trials), alternating them in a quasi-random order. In the test trials, less than 15% of the animals remained on the mat. Dogs reliably faster left the mat in the test trials than in all three types of controls. They left the mat reliably slower in the tests when the stick was tied to the hook, and more often they did this by releasing the stick, while in the test trials they more often left the mat without releasing the stick. In the control conditions in which the experimenter pulled on the rope tied to the mat, the dogs stayed on it significantly more often than in the test trials, which indicates that the sensation of tissue tension under the legs is not enough for the dog to get off the mat. Overall, these results indicate that dogs understand the structure of this task and have an idea that their body has weight.

In addition to body weight awareness, children, and dogs were further studied on their body size awareness, i.e., their ability to correlate their own body size with the size and shape of the hole through which they had to pass was evaluated. In working with children (Brownell et al., 2007), in order to reach their parent, children had to choose between two holes in the partition: one was unsuitable for passage (high and narrow, 10 × 80 cm), and the second one was suitable (30 × 30 cm). Before each test, the parent peeked through each hole several times and attracted the child’s attention. Then the parent sat down on a chair and called the child, who at that time, together with the experimenter, was on the other side of the partition at the same distance from both holes. As soon as the child passed through the hole and approached the parent, the experimenter called the child back. Thus, each child had to choose a hole to pass through twice. Eighteen-month-old children chose a passable hole only after one or more mistakes. At 22 months, the number of errors was significantly reduced, and at 26 months, some children chose a passable hole without prior tactile contact with it (Brownell et al., 2007). This suggests that the ability to mentally operate with ideas about the size of own body, like the idea that your own body has weight, manifests itself closer to the third year of life. At about the same time, at the age of 18–24 months, children begin to recognize their reflection (Bertenthal and Fischer, 1978). The emergence of the ability to recognize one’s own reflection correlates with the emergence of the ability to understand the needs and intentions of other people (Bischof-Köhler and Bischof, 2018).

With dog subjects (Lenkei et al., 2020), a different technique was used. The animals did not have to choose between two holes. The authors evaluated the latency period for approaching a single hole in the partition by changing its size. In the first experiment, 12 conditions were quasi-randomly alternated between suitable and unsuitable for passage holes. In the last 13 test trials, the hole was passable, but smaller than the larger of the two previously used. The latency period of approach to it was compared with the results of the last two trials (eleventh and twelfth), in one of which the hole was unsuitable for passage, and in the other it was suitable. It turned out that during the 13th trial all dogs approached the hole significantly slower than the previously used large hole, but significantly faster than the small hole unsuitable for passage. In the second experiment, the hole size was reduced from trial to trial. It turned out that the latency period of the approach increased insignificantly until the size of the hole was reduced to a certain value, at which it either became really unsuitable for passage, or so small that the animal would have to crawl through it, and in this case all 32 the dogs did not even try to approach it. After that the size of the hole was increased by “one step” and the dogs again began to pass through it. This result may indicate that dogs can correlate the idea of the size of their body with the size of the hole. The third experiment explored whether the dog’s anatomical peculiarities influenced the dog’s latency to pass. Four tests were performed on either long-legged dogs of different breeds or welsh corgis. In the first three, rectangular holes 60 cm high were used, the width of which was equal to the height at the withers of the lying dog. In the fourth test, a hole of the same size was used, but oriented with its long side horizontally. In all dogs, regardless of whether they were long or short legged, the latency period for approaching this particular hole was longer. Thus, this work provides a second confirmation that dogs have some degree of body awareness development. These data are consistent with the fact that dogs have elementary theory of mind, e.g., the ability to assess the focus of a person’s attention. They more often begged for food from people who were looking at them and performed prohibited actions when the person did not look at them (Udell et al., 2011). There is no reliable evidence yet that dogs can recognize their reflection (Lenkei et al., 2020). However, Howell and Bennett (2011) investigated the ability of dogs to understand the nature of reflection: 2 of 40 dogs tested appeared to be able to understand the actual location of their owner based on the information provided in the reflection.

The studies of the features of body-awareness in animals are conducted as an addition to the classic mirror test. The phenomenon is considered as the ability of animals to take into account various characteristics of their own body (such as size and weight) as obstacles to achieving the goal. On the one hand, they complement the data on the specificity of self-awareness in those animals that demonstrate self-recognition in the mirror (Dale and Plotnik, 2017). On the other hand, these studies reveal features of self-awareness in those animals that usually fail the mirror test (Lenkei et al., 2020, 2021). Consequently, the methods of studying body-awareness enrich our knowledge about the specificity of self-awareness in different animal species.

The main difficulty in studying body-awareness is to exclude the effect of learning. This is achieved either by recruiting a large number of subjects with 1–2 tests on each (Brownell et al., 2007; Lenkei et al., 2021), or by comparing background and test trials if a small number of subjects is available (Dale and Plotnik, 2017).

The results of numerous independent studies using different experimental approaches show that corvids achieve cognitive feats that are comparable to those exhibited by primates (Smirnova et al., 2015, 2021; Emery, 2016; Güntürkün and Bugnyar, 2016; Güntürkün et al., 2017). Corvids’ remarkable cognitive abilities are based on the high level of their brain complexity (Emery, 2016; Olkowicz et al., 2016). These birds have an encephalization quotient comparable to that of monkeys and apes, and larger than any other bird group (Sayol et al., 2016). Brains of these birds harbor absolute numbers of neurons that are comparable, or even larger than those of primates with much larger brains (Olkowicz et al., 2016). Parrots and corvids have much higher proportions of brain neurons located in the pallial telencephalon compared with other birds (Olkowicz et al., 2016).

It is also important to pay attention to the linkage between ontogeny and cognitive performance of corvids. A 2020 study using the Primate Cognition Test Battery (PCTB) demonstrated that full-blown cognitive skills are found as early as 4 month old ravens and do not change at later ages (Pika et al., 2020).

Corvids are able to track the goals, perceptions, and knowledges that motivate others’ actions (Emery and Clayton, 2001; Dally et al., 2006, 2010; Schloegl et al., 2009; von Bayern and Emery, 2009; Bugnyar et al., 2016; Emery and Clayton, 2016; Ostojić et al., 2016, 2017). Data on their ability to recognize their reflection is contradictory. Along with the results indicating that some corvids (magpies, Prior et al., 2008; Clark nutcrackers, Clary and Kelly, 2016; Indian house crows, Buniyaadi et al., 2019) can recognize their reflection, negative results were also obtained (Soler et al., 2014, 2020; Smirnova et al., 2019; Vanhooland et al., 2019; Brecht et al., 2020; Clary et al., 2020; Parishar et al., 2021). The degree of development of understanding the physical properties of their bodies in corvids and other birds has not been previously studied.

The goal of the current work was to investigate whether hooded crows (*Corvus cornix*) may be aware of their own body size.

Below we present the results of two experiments in which the crows had to choose which of the three holes to go through. In the first experiment, we tried to find out whether the crows preferred a larger hole if all three are suitable for passage, and what other predictors influence their choice. The second experiment assessed the ability of the crows on the first attempt to select a single passable hole out of three, even though its area was smaller than the other two. In doing this we tried to exclude alternative decision-making mechanisms, such as simple preference for the more conveniently sized opening, relying on learning about the suitability of a particular hole, or on an *a priori* experience with holes of various size and shape.

## Materials and Methods

### Subjects

A total of six hooded crows (*Corvus cornix*) were used for these experiments. All birds were housed in the outdoor aviary of the Biology Department of Lomonosov Moscow State University, Russia. The crows were wild-caught and, after treatment in a veterinary hospital due to getting injured in the wild, had been kept for about 1–10 years prior to these experiments. The birds were kept in groups in the open-air enclosure (230 × 350 × 280 cm). All birds were identified by a number on a metal leg ring and had not been used for any other experiments in which they were required to pass through an opening in a wall.

Since we used a new technique not previously adapted for birds, all experiments were conducted first with one group of birds, and then with a second group, for which the procedure was slightly adjusted. We divided the crows according to the age criterion: the first group included sub-adults and the sexually mature ones constituted the second group. In the first group, all three crows (“Joe,” “Rodya,” and “Dyatel”) were older than one, but no older than 2 years old, while in the second group one bird, “Malyschka” was 4 years and two birds, “Glaz” and “Schnobel” at least 10 years old. We assumed that the age of an animal might influence the cognitive phenomenon under study. Specifically, in this study we assumed that adult birds would make fewer mistakes in choosing a passable hole.

### Experimental Setup

**FIGURE 1:**
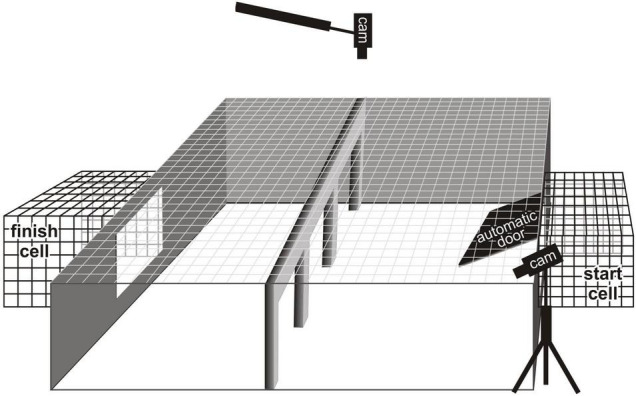
The experimental setup and location of video cameras.

The experimental setup (Figure 1) consisted of a rectangular arena (110 × 130 × 40 cm) with opaque walls, which was divided into two halves by a vertical opaque Plexiglas panel (110 × 40 cm) with three openings. The arena was covered with a metal lattice with the size of cells 5 × 5 cm.

The presence/absence, size and shape of the holes could be adjusted with additional plates inserted into the grooves on their sides. Square holes (30 × 30 cm) were made in the middle of the two opposite walls of the arena. To one of them, equipped with a remotely opening door, the “start” cell might be attached (the cage in which the bird was placed after catching played the role of the “start” cell). A “finish” cell with a feeder was placed at the other hole in the wall, into which three flour beetle larvae were placed.

When working with the first group of birds, the installation was placed in such a way that the “start” cell was opposite to the door of the room. In this case, to the right of the installation was the wall of the room, and to the left there was a free space along which the experimenter moved during preparation for the test.

In order to reduce the influence of the inhomogeneity of the surrounding space when working with the second group of birds, the installation was moved to the far part of the room and fenced off with a white cloth from all sides (from floor to ceiling).

**FIGURE 2:**
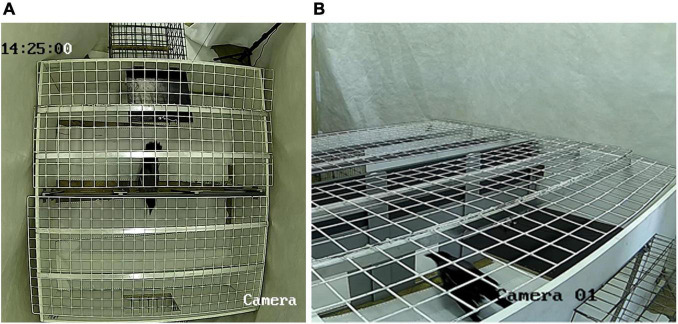
The experimental setup configured for the second group of crows as viewed from the camera 1 **(A)** and camera 2 **(B)**.

The video registration system included two EZVIZ C2W cameras connected to an Ezviz Vault Live CS-X5C-4EU video recorder. Monitoring and control of the recording was carried out using the EZVIZ Studio 2.1.1.0 program. One camera was placed above the center of the arena, and the second was located near the left corner on the side of the “start” cell (Figure 2).

### General Procedure

The birds were transferred to the experimental room and placed in individual cells of 40 × 40 × 40 cm with a drinker with water and a feeder with three mealworm larvae. The experiment was started at least 15 min later.

The experimenter placed a “start” cell with a crow to the opening leading into the arena, which was closed with a door, and left the experimental room. From another room, the experimenter opened the door separating the “start” cell and the arena and observed the behavior of the crow on the monitor screen.

The test was considered completed if the bird passed through the hole in the partition and entered the “finish” cell in no more than 5 min. If the bird did not pass through the hole for 5 min, the trial was interrupted. In both cases, the “finish” cell together with the crow was set aside and the next test was carried out with another crow. No more than 9 trials were allocated to each bird per day.

If the birds refused to go through the openings, then the proportion of meat in their diet was reduced for 1 or 2 days.

Two experiments were carried out, in each of which the birds were first trained to pass through smaller holes and then the test was performed.

### Experiment 1

In the first experiment, we studied whether the birds would prefer to get through a larger hole if the other two were also suitable for passing.

#### Training

Initially, birds from both groups were trained to pass through a single opening in the partition and enter the “finish” cell. The largest hole (30 × 30 cm) was used, the location of which was changed quasi-randomly in each subsequent trial, i.e., the same hole was open for no more than two sequential trials, and each of the holes was open an equal number of times (three times each). Nine tests were carried out with each crow.

Then all the birds were taught to pass through a single smaller hole. The whole location was changed quasi-randomly in each subsequent trial. For birds from the first group, a rectangular hole was used, oriented vertically with its long side (10 × 18 cm). Initially, we did not plan to use holes oriented horizontally, therefore we did not use them when training the crows of the first group. Eighteen trials were performed by each of the three birds.

For birds from the second group, in half of the trials, rectangular holes were oriented with their long side vertically (10 × 20 cm), and in the other half, horizontally (20 × 10 cm). These two types of conditions alternated quasi-randomly. Twelve trials were performed by each of the three birds.

We changed the conditions in the second group believing that the difference in the shape and size of the rectangular holes 10 × 20 cm and 20 × 10 cm would be more noticeable for the crows. In the first group, by the 12th trial, the crows solved the problem in less than 1 min, and then this indicator did not decrease, so in the second group we reduced the number of experimental trials from 18 to 12.

#### Testing

The partition had three rectangular holes, one of which was larger in area than the other two. It was either wider when all three holes were oriented with the long side vertically, or higher when all three holes were oriented with the long side vertically. The location of the larger hole was changed quasi-randomly.

**FIGURE 3:**
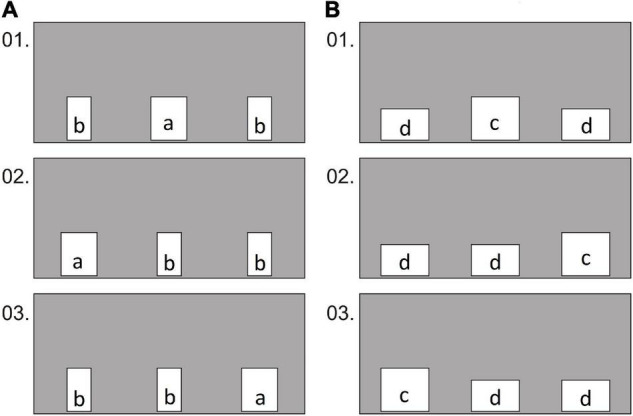
Experiment 1, group 1, test: examples of the first 3 conditions in which the holes were oriented with the long side either vertically **(A)** or horizontally **(B)**. Hole sizes: a—15 × 18 cm; b—10 × 18 cm; c—20 × 18 cm; d—20 × 13 cm.

**FIGURE 4:**
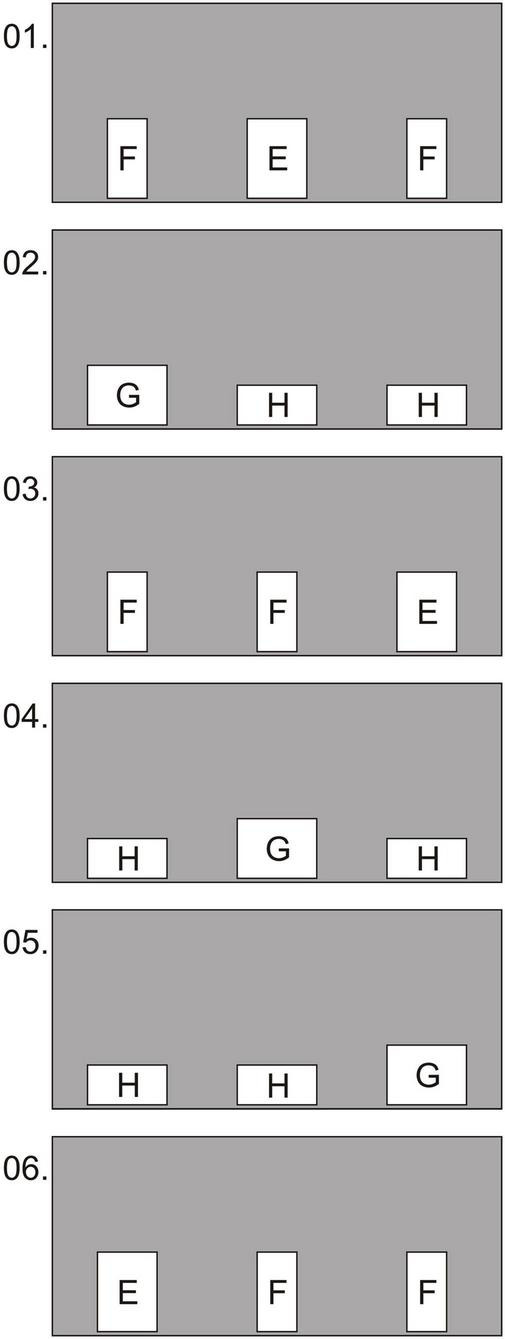
Experiment 1, group 2, test: examples of the first 6 conditions in which the holes were oriented with the long side either vertically (conditions 1, 3, 6) or horizontally (conditions 2, 4, 5). Hole sizes: E—15 × 20 cm; F—10 × 20 cm; G—20 × 15 cm; H—20 × 10 cm.

The first group of birds was first tested in 36 trials (Figure 3A), in which the holes were different in width, one was wider (15 × 18 cm) than the other two (10 × 18 cm). Then 36 trials were made with them (Figure 3B), in which the holes differed in height, one (20 × 18 cm) was higher than the other two (20 × 13 cm). With the second group of birds, these two types of conditions alternated quasi-randomly (Figure 4). In half of the 72 trials, the larger hole was wider (15 × 20 cm) than the other two (10 × 20 cm). In the other half of the conditions, the larger hole (20 × 15 cm) was higher than the other two (20 × 10 cm).

#### Statistical Analysis

In each trial, two indicators were recorded: the first approach to the hole and the passage through the hole. The approach was considered a situation when the distance between the tip of the bird’s beak and the hole was no more than 10 cm.

The statistical analysis was conducted using Statsoft Statistica, version 10.0.1011.0.

In total, for all six birds, the presence/absence of a connection between the first approach to one of the holes and the passage through it was assessed. To do this, we compared the empirical distribution of the total number of approaches to holes, followed by passing through the same hole, and the number of approaches after which birds passed through another hole, with a hypothetical uniform distribution (50%/50%) using Pearson’s chi-squared test (χ^2^).

The factorial ANOVA was carried out jointly for birds from both groups (*n* = 6). The following were used as predictors: group number (1 and 2), position of the opening (right/central/left), area of the opening (larger/smaller), hole orientation (vertical/horizontal). The number of passages through the holes was used as the response variable. Two-way interactions between the factors were taken into account (three-way and four-way interactions were not identified in any of the cases; therefore, they were not included in the model). Subject ID was included as a random factor. The effect of the differences between the levels of the factors was determined using Tukey’s *post hoc* test. The model was also checked for the uniformity of the distribution of errors and the absence of collinearity between predictors.

### Experiment 2

In the second experiment, it was ascertained whether birds would choose a passable hole if it was smaller in area than the other two unsuitable for passage.

#### Training

Initially, all birds were taught to pass through a single hole in the partition (its location was changed quasi-randomly), gradually decreasing its size: first, holes 10 × 14 cm were used, then 10 × 12 cm, and finally 10 × 10 cm. The latter were minimally passable to the crows, since their size was comparable to the body size of the birds. With each of the first two holes 9 trials were made. With a hole of the minimum size, from 12 to 21 trials were made, ensuring that the crows passed through it in no more than 5 min.

#### Testing

**FIGURE 5:**
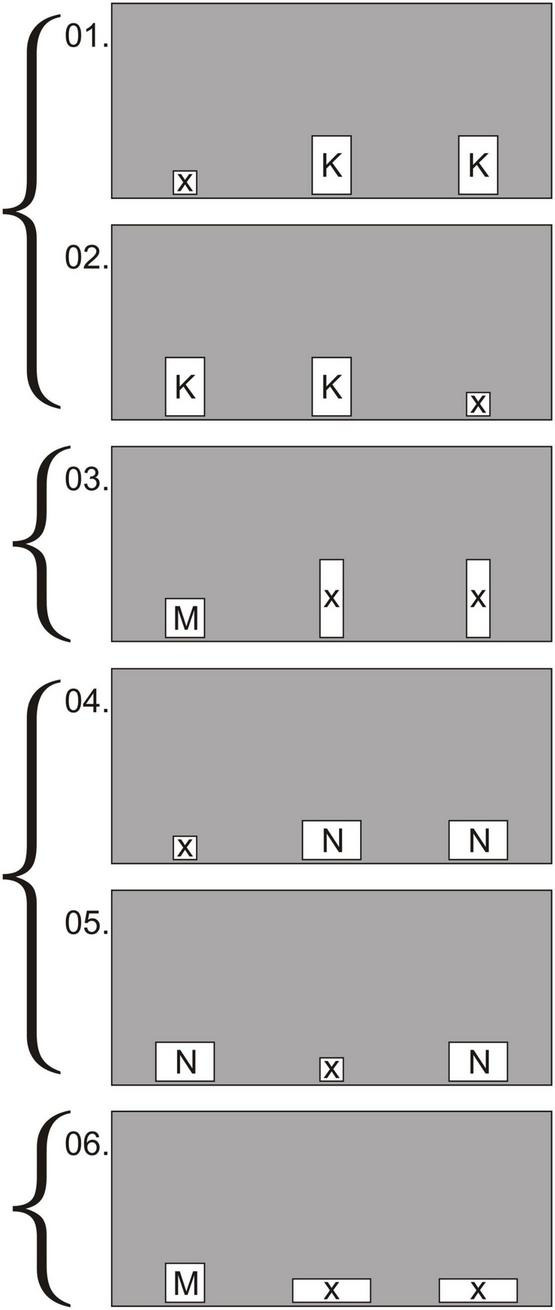
Experiment 2, test: example of the first 6 trials out of 36. 1, 2, 4, and 5 are background trials; 3 and 6—test trials. Holes K, M, and N suit for passage: K—10 × 15 cm; M—10 × 10 cm; N—15 × 10 cm. Holes X unsuitable for passage—6 × 6 cm, 6 × 20 cm, 20 × 6 cm.

There were three holes in the partition. Test and background trials were alternated (Figure 5). In the test conditions, the passable square hole (10 × 10 cm) was smaller in area than the two unusable ones. In half of the trials, these holes were oriented vertically (6 × 20 cm), and in the other half, horizontally (20 × 6 cm). The location of the square hole suitable for passage was changed quasi-randomly. Each test condition was presented after two background ones, in which a square hole (6 × 6 cm) was unsuitable for passage, in contrast to two rectangular (10 × 15 cm; in half of the conditions they were oriented vertically, and in the other half horizontally). The purpose of alternating background and test conditions was to exclude the effect of learning to pass through holes of a certain shape (squares 10 × 10 cm), since in the background conditions the squares were non-passable, and the rectangles were passable. The location of the unsuitable square hole was changed quasi-randomly. We conducted 12 test and 24 background trials.

#### Statistical Analysis

The results of 12 test trials were analyzed. In each test trial, two indicators were recorded, the first approach to the hole and the first attempt to enter the hole. The approach was considered a situation when the distance between the tip of the bird’s beak and the hole was no more than 10 cm. An attempt was a penetration in which at least half of the crow’s head was on the opposite side of the hole. This was considered an attempt at a passage, not a passage, since the attempt could be made into an impassable opening. Precisely these indicators were considered and analyzed, since we were interested in the choice of the hole made without feedback from the interaction with the walls of the hole.

For each bird, the reliability of differences from the random level (33.3%) was assessed in the total number of first approaches to the hole suitable for passage, as well as attempts to enter it (binomial test). Since our sample consisted of only 5 birds (one crow was excluded from the experiment—see the explanation in the “Results” section), we calculated the effect size for the number of first approaches to the holes in the trial and the first attempts to pass through the holes. We used Cohen’s d as an effect size measurement [*d*-value of 0.20 indicates a small effect size, 0.50 indicates a medium-sized effect, and 0.80 indicates a large effect (Cohen, 1988)].

Further analysis was performed for all five birds in total. To identify the presence/absence of a connection between the first approach to one of the holes and an attempt to pass through it, we compared the empirical distribution of the total number of approaches to the holes, followed by an attempt to pass through the same hole, and the number of approaches after which the bird attempted to pass through another hole, with a hypothetical uniform distribution (50%/50%) using Pearson’s chi-squared test (χ^2^).

The presence/absence of the same relationship was then analyzed separately for the first approaches to passable and non-passable openings using linear regression.

Factorial ANOVA was carried out for birds from both groups (*n* = 5). The following were used as predictors: group number (1/2), hole passability (passable/non-passable), hole position (right/center/left), hole area (larger/smaller), orientation of the non-passable opening (vertical/horizontal). The following were used as dependent variables: the number of first approaches to the holes in the trial, the number of first attempts to pass through the holes in the trial. Two-way interactions between the factors were taken into account (three-way and four-way interactions were not identified in any of the cases; therefore, they were not included in the model). Subject ID was included as a random factor. The effect of the differences between the levels of the factors was determined using Tukey’s *post hoc* test. The model was also checked for the uniformity of the distribution of errors and the absence of collinearity of predictors.

Using the Friedman test, it was determined whether the number of attempts to pass through the non-suitable and suitable holes changed during the 12 test trials.

## Results

### Experiment 1

The summary of the results of all six birds showed that crows significantly more often pass through the hole they first approached, in 363 out of 432 cases (χ^2^ = 113.142; df = 1*; P* < 0.001).

In both groups of birds, the size of the hole (larger/smaller), and its position (right/center/left) were predictors influencing the number of passes.

**FIGURE 6:**
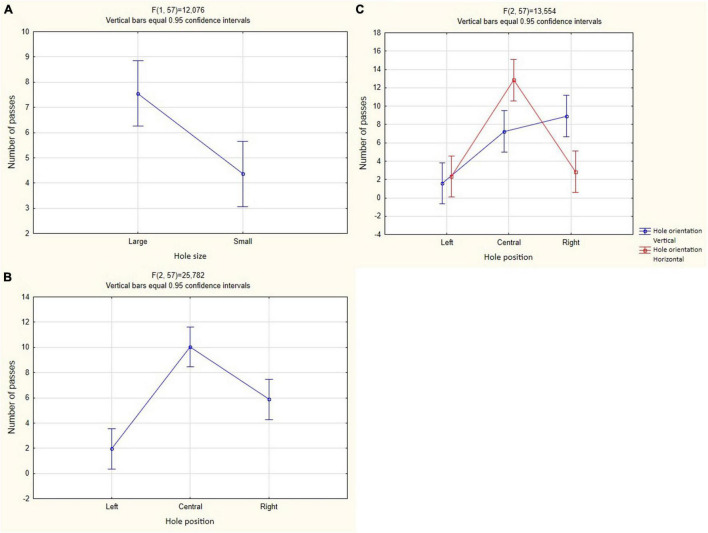
Mean and 95% confidence intervals for the number of passes through holes in Experiment 1 in both groups (*N* = 6). **(A)** Is the predictor of the hole size; **(B)** hole position predictor; **(C)** mutual influence of predictors of hole orientation and hole position.

Birds more often passed through a larger hole [*F*_(1_, _57)_ = 12.076, *P* < 0.001; Figure 6A and Table 1]. These crows more often used the central opening for the passage than the left one [*F*_(2_, _57)_ = 25.782, *P* < 0.001; Tukey’s *post hoc* test, *P* < 0.01; Figure 6B and Table 1] and the right one [*F*_(2_, _57)_ = 25.782, *P* < 0.001; Tukey’s *post hoc* test, *P* < 0.01; Figure 6B and Table 1]. Also, crows used the right hole more often than the left one [*F*_(2_, _57)_ = 25.782, *P* < 0.001; Tukey’s *post hoc* test, *P* < 0.01; Figure 6B and Table 1].

**TABLE 1:** Results of the Experiment 1: evaluation of the influence of various predictors (group number, position, size, and orientation of the holes) on the number of passes, the factorial ANOVA.

Predictor	SS	df	MS	*F*	*P*
Group number	0.125	1	0.125	0.008	0.928
Hole orientation	0.125	1	0.125	0.008	0.928
Hole size	183.681	1	183.681	12.076	0.001
Hole position	784.333	2	392.167	25.782	0.001
Group number*Hole orientation	0.125	1	0.125	0.008	0.928
Group number*Hole size	0.347	1	0.347	0.023	0.880
Hole orientation*Hole size	30.681	1	30.681	2.017	0.161
Group number*Hole position	52.333	2	26.167	1.720	0.188
Hole orientation*Hole position	412.333	2	206.167	13.554	0.001
Hole size*Hole position	13.778	2	6.889	0.453	0.638

#### Joint Effect of Predictors

In cases where the holes differed in width rather than height, these crows more often passed through the central hole [*F*_(2_, _57)_ = 13.554, *P* < 0.001; Tukey’s *post hoc* test, *P* < 0.05; Figure 6C and Table 1]. On the other hand, in cases where the holes differed in height rather than width, these crows more often passed through the right hole [*F*_(2_, _57)_ = 13.554, *P* < 0.001; Tukey’s *post hoc* test, *P* < 0.01; Figure 6C and Table 1]. The effect of other predictors on the number of passes was not found.

### Experiment 2

In the second group, one of the crows (“Malyschka”) refused to leave the “start” cell from the 4th test trial, so from that point on the results of the second group were obtained only for two birds.

The summary of the results of all five birds showed that during the 24 background trials, the crows significantly more often passed into the same hole to which they made the first approach, i.e., in 104 cases out of 120 (χ^2^ = 37.279; df = 1; *P* < 0.001). In contrast to the background, in 12 test trials we did not reveal a significant connection between the first approach and the first attempt to enter the same hole: only in 38 cases out of 60 crows entered the hole which they first approached (χ^2^ = 2.172; df = 1; *P* = 0.141).

**FIGURE 7:**
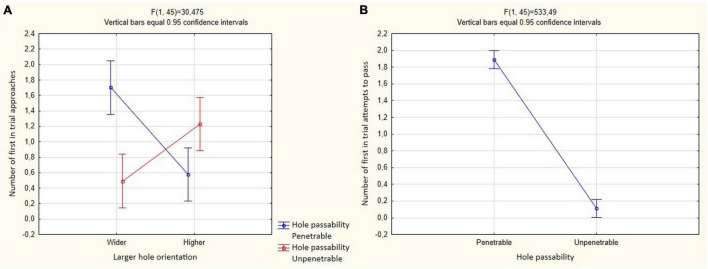
Mean and 95% confidence intervals for the number of passes through holes in Experiment 2 of 12 test trials in both groups (*N* = 5). **(A)** Mutual influence of predictors of hole passability and large hole orientation, dependent variable is the number of first approaches to the holes in the trial; **(B)** hole passability predictor, dependent variable is the number of first attempts to pass through the holes in the trial.

The birds more often performed the first approach to a passable hole only if it was higher than the unsuitable one [*F*_(1_, _45)_ = 30.475, *P* < 0.001; Tukey’s *post hoc* test, *P* < 0.01; Figure 7A and Table 2]. The effect of other predictors on the first approach to a hole was not found.

**TABLE 2:** Results of the Experiment 2 of 12 test trials: evaluation of the influence of various predictors (group number, passability of the hole, position, orientation of the larger hole) on first approaches in a trial, the factorial ANOVA.

Predictor	SS	df	MS	*F*	*P*
Group number	0	1	0	0	1
Hole passability	1.111	1	1.111	2.591	0.114
Hole position	0.517	2	0.258	0.603	0.552
Larger hole orientation	0.544	1	0.544	1.270	0.266
Group number*Hole passability	1.111	1	1.111	2.591	0.114
Group number*Hole position	0.517	2	0.258	0.603	0.552
Hole passability*Hole position	0.133	2	0.067	0.155	0.856
Group number*Larger hole orientation	0.011	1	0.011	0.026	0.873
Hole passability*Larger hole orientation	13.067	1	13.067	30.475	0.001
Hole position*Larger hole orientation	1.200	2	0.6	1.399	0.257

In test trials, the first attempt to pass through a passable hole did not always result in a pass. Birds often made several attempts to pass through it, and only then did they pass. Accordingly, during one test, crows could make several attempts to pass through different holes. On the other hand, there were cases when crows made several attempts to enter an impassable opening. So, the “Dyatel,” in the sixth trial, approached an unsuitable hole and tried to pass through it 33 times.

However, regression analysis found that crows significantly more often attempted to enter the hole they approached first if it was suitable for passage (*R* = 0.975; *B* = 0.937; *P* < 0.005). When the birds approached a hole unsuitable for passing, such a pattern was not revealed (*R* = 0.058; *B* = 0.062; *P* = 0.926).

Meanwhile, in some birds during 12 test trials, a significant predominance was revealed of the first approach to a passable hole followed immediately by passing through the hole (without approaches to any other hole). In the first group, two out of three birds (“Joe” and “Dyatel”) a significantly more frequent first approach to the passable hole was followed by passing through it. (9/12; *P* = 0.003 and 8/12; *P* = 0.015; binomial test). “Rodya” made the first approach to the passable hole and then immediately passed through it only in 6 out of 12 trials (6/12; *P* = 0.108; binomial test).

In the second group, one of the two remaining birds (“Schnobel”) significantly more often approached the passable hole first and then passed through it (7/12; *P* = 0.047; binomial test). “Glaz” made the first approach to the permeable hole and then immediately passed through it only in 5 out of 12 trials (5/12; *P* = 0.188; binomial test).

At the same time, the effect size for the number of the first in trial approaches to the holes for five subjects turned out to be large (Cohen’s *d* = 0.963; *n* = 5; *M* = 7, SD = 1.58).

Below is the analysis of the number of attempts to pass made by the birds during 12 test trials.

Each of the five birds reliably more often made their first attempt to pass through a hole suitable for this: “Joe” and “Rodya”—12/12; *P* = 0.001; “Dyatel,” “Schnobel” and “Eye”—11/12; *P* = 0.001 (binomial test).

At the same time, the effect size for the number of the first in trial attempts to pass through the holes for five subjects turned out to be large (Cohen’s *d* = 1; *n* = 5; *M* = 11.4, SD = 0.55).

Birds significantly more often made the first attempts to pass through holes suitable for this [*F*_(1_, _45)_ = 533.488; *P* < 0.001; Figure 7B and Table 3]. The effect of other predictors on the first attempt to pass was not found.

**TABLE 3:** Results of the Experiment 2 of 12 test trials: assessment of the influence of various predictors (group number, passability of the hole, position, orientation of the larger hole) on the number of first attempts to pass in a trial, the factorial ANOVA.

Predictor	SS	df	MS	*F*	*P*
Group number	0	1	0	0	1
Hole passability	45.511	1	45.511	533.488	0.001
Hole position	0.417	2	0.208	2.442	0.098
Larger hole orientation	0.1	1	0.1	1.172	0.285
Group number*Hole passability	0.178	1	0.178	2.084	0.156
Group number*Hole position	0.017	2	0.008	0.098	0.907
Hole passability*Hole position	0.4	2	0.2	2.344	0.108
Group number*Larger hole orientation	0.1	1	0.1	1.172	0.285
Hole passability*Larger hole orientation	0.267	1	0.267	3.126	0.084
Hole position*Larger hole orientation	0.133	2	0.067	0.781	0.464

During 12 test trials, the number of first attempts to enter a hole suitable for passing did not change significantly (Friedman test = 13.245, *n* = 5, cc = 11, *P* = 0.278).

## Discussion

### Discussion of the Results of Experiment 1

The results of Experiment 1 demonstrate that the crows of both groups preferred to pass through larger openings. Moreover, the birds usually passed through the hole which they first approached.

However, the choice of the hole was also influenced by its position (left, middle, right). In addition, the combined influence of the predictors of the position and orientation of the holes, as well as the position and size of the holes was revealed.

In conditions when the holes were equally high and differed only in width, the crows preferred to pass through the central one, i.e., chose the shortest way to the “finish” cell. If the holes were equally wide and differed only in height, then there was no preference for the central one.

Avoidance of the left opening was observed in all birds. Perhaps this is because, both in the experiments with the first and in the second group, the experimenter was moving along the left side of the experimental setup during the preparation of the trial and after its completion. Also, this could be due to the fact that one of the cameras was installed on the left side of the experimental setup. It is important to note that the group number (1 or 2) did not predict the choice of the hole for passage. Accordingly, the choice of the hole was not influenced by differences in the organization of experimental trials and the type of arrangement of the experimental setup in the room, neither by differences in the age of the birds.

The fact that in both groups the birds preferred to pass through the central hole when the holes differed in width (rectangles with the long side oriented vertically) may signal that it was equally convenient for them to pass through both narrow and high holes as well as through wide and high holes, and the major predictor in this case was the choice of the shortest path. When choosing among holes that differ in height (rectangles with the long side oriented horizontally), they preferred to go through the higher one, probably because for this they did not need to squat and make other additional movements. This is consistent with evidence that dogs were slower to approach lower holes (Lenkei et al., 2020).

### Discussion of the Results of Experiment 2

First, it should be noted that, during 24 background trials the birds usually passed through the hole which they approached first. In contrast to background trials, we did not find a significant relationship between the first approach and the first attempt to pass through the same hole during 12 test trials. Only if the hole which the birds first approached was suitable for passage, they made the first attempt to pass through it significantly more often. Although in 12 test trials the birds performed the first approach to the passable hole only if it was higher than the non-passable one.

We believe that when approaching the hole, the crows determined whether the hole was passable or not. Accordingly, given that in the background probes the crows significantly more often performed the first approach followed by passage through a suitable hole, the task in the test trial (when it was necessary to choose between small passable and large unpassable holes) turned out to be more difficult for birds. During the 12 test trials, not all birds approached the passable hole first. However, “Joe,” “Dyatel,” and “Schnobel” significantly more often approached the passable hole first and then passed through it during the test trials.

Despite the fact that only three out of 5 crows reliably more often carried out the first approach and then immediately the first attempt to pass, all crows reliably more often made their first attempt to pass through a hole suitable for passing, even if the first approach in the trial was carried out to the non-passable hole. In contrast to the first experiment, no influence of the hole orientation and its position on the choice was revealed.

It is important to note that since the number of first attempts to enter a suitable hole did not change reliably during 12 test trials, we can argue that the choice of a suitable hole for passing was not a result of learning during the test trials of the second experiment. Thus, the method we used for organizing the test trials when they followed two background ones, in which the hole was unsuitable for passage, turned out to be effective.

In addition, it should be noted that a greater number of first attempts to pass through the passable holes, as well as a greater number of first approaches to the passable holes followed by passage (three birds—“Joe,” “Dyatel,” and “Schnobel”) was not conditioned by learning to penetrate precisely into holes of exact shape and size (squares 10 × 10 cm) formed during training before Experiment 2. This thesis is substantiated by the fact that in 24 background trials the birds significantly more frequently (in 104 cases out of 120) made the first approach and subsequent passage through the passable hole while in these trials the passable hole was of different shape and size.

As mentioned above, the orientation of the holes did not affect the number of first attempts to pass through them. However, as in the first experiment, it influenced the number of first approaches. All crows more often made the first approach to a passable square hole only if it was higher than an unusable rectangular one. Thus, birds were attracted primarily by the higher holes, since passing through them required less effort.

One should also consider the possibility that the first approaches and first attempts to pass in 12 test trials were influenced by the training that preceded the test trials in the Experiment 2. In 12 test trials, the birds had to choose between one familiar hole through which they passed earlier, and two unfamiliar holes. This must be taken into account in the organization of further similar experiments on animals in order to exclude the undesirable effect of learning, since the aim of training was passing through the holes in general, and the objective of the study was selecting a passable hole thus demonstrating body awareness.

Additionally, one should note that, as in the Experiment 1, in the Experiment 2 the group number (1 or 2) was not a predictor of hole selection for the first approach or first passage. In this study we assumed that adult birds would make fewer mistakes in choosing a passable hole. However, this influence was not detected. This is probably due to the fact that full-blown cognitive skills are formed in corvids by the age of 4 months and do not change significantly at the age of 8, 12, and 16 months (Pika et al., 2020). All our crows were over a year old. Accordingly, to identify the age-related dynamics of body-awareness development in corvids, one should compare adult birds with fledglings (younger than 4 months old). Also, a possible factor in the lack of differences between the two experimental groups was the small sample size.

## Conclusion

In general, the results obtained showed that despite the presence of a preference for a larger hole in area, revealed in the first experiment, in the second experiment, taking into account both the first attempts to pass through the hole and the first approaches to the hole followed by passage through the hole, it should be concluded that at least 3 out of 6 crows (“Joe,” “Dyatel,” and “Schnobel”) were able to choose a hole suitable for passage, even if its area was smaller than that of an unsuitable one.

Since we took into account the first in trial approach to a hole and the first attempt to pass through one, we can argue that the birds chose the hole suitable for the passage by comparing its size with ideas about the size of their body, and not due to feedback from touching the walls of the hole or whatever else. Considering the large effect size both for first approaches and first attempts to pass, it can be argued that the hooded crows may develop the ability to aware of their own body size.

However, we believe it is necessary to conduct additional studies to determine precisely whether the choice by the crows of a suitably sized hole for passage is the evidence of their body awareness. In particular, as noted above, it is necessary to exclude the undesirable effect of learning, since the aim of training was passing through the holes in general, and the objective of the study was selecting a passable hole thus demonstrating body awareness. In addition, this will reveal the evolutionary diversity of cognitive mechanisms that ensure the solution of problems involving taking into account the size of one’s own body.

For example, using a similar technique, it was found that snakes *Elaphe radiata* are not able to select a hole suitable for passage on the first try, i.e., by comparing its size with ideas about the size of their body, but they are able to learn this after dozens of trials (Khvatov et al., 2019). Also, studies conducted on hermit crab have demonstrated that these invertebrates are capable of taking into account their body size, as well as the modified structure of the shells (Sonoda et al., 2013; Krieger et al., 2020).

Our results are consistent with data on the high level of brain development and cognitive abilities of corvids (Smirnova et al., 2015, 2021; Emery and Clayton, 2016; Güntürkün and Bugnyar, 2016; Güntürkün et al., 2017). It is important to note that other aspects of consciousness have been previously revealed in them: the capacity to infer mental states in others (Emery and Clayton, 2001, 2016; Bugnyar and Heinrich, 2005; Dally et al., 2006, 2010; Schloegl et al., 2009; von Bayern and Emery, 2009; Bugnyar et al., 2016; Ostojić et al., 2016, 2017), the capacity for self-recognition (Prior et al., 2008; Clary and Kelly, 2016; Buniyaadi et al., 2019), episodic-like memory for past events (Clayton and Dickinson, 1998; Clayton et al., 2003; Clayton and Emery, 2015), and episodic-like planning of future events (Raby et al., 2007; Cheke and Clayton, 2012).

To date, signs of body size awareness have been found in children aged 22–26 months (Brownell et al., 2007) and dogs (Lenkei et al., 2021). The methodology we have used expands the set of tests applicable to body-awareness research. It can be applied to a wide range of species, which will allow tracing the development of this cognitive ability in phylogeny.

Expanding the set of tests used and advances in methods, as well as improved coverage of the species studied, can help clarify the cognitive architecture of self-awareness (De Waal, 2019).

## Data Availability Statement

The original contributions presented in the study are included in the article/Supplementary Material, further inquiries can be directed to the corresponding author/s.

## Ethics Statement

The animal study was reviewed and approved by the Biology Department of Lomonosov Moscow State University, Russia.

## Author Contributions

IK: conceptualization, formal analysis, data curation, and project administration. AS, IK, and MS: methodology. EE: software, resources, and visualization. EE and AS: validation. MS, SB, and EE: data collection. IK and AS: writing, original draft preparation. AK: writing, review and editing and supervision. All authors contributed to the article and approved the submitted version.

## Conflict of Interest

The authors declare that the research was conducted in the absence of any commercial or financial relationships that could be construed as a potential conflict of interest.

## Publisher’s Note

All claims expressed in this article are solely those of the authors and do not necessarily represent those of their affiliated organizations, or those of the publisher, the editors and the reviewers. Any product that may be evaluated in this article, or claim that may be made by its manufacturer, is not guaranteed or endorsed by the publisher.
