# Supplementary material for: Hooded Crows (Corvus cornix) May Be Aware of Their Own Body Size
Source: Front Psychol. 2021 Dec 16;12:769397. doi: 10.3389/fpsyg.2021.769397 (PMC8716556; doi:10.3389/fpsyg.2021.769397)
Supplement: Supplementary file 1 [file Table_1.DOCX]

Supplementary Material

# Table 1. The number of passes through the holes in group 1 in experiment 1

| Crow | Tipe of the hole | | | Number of passes |
| --- | --- | --- | --- | --- |
|  | Hole orientation | Hole size | Hole position |  |
| Rodya | Vertical | Large | Left | 1 |
|  | Vertical | Large | Central | 10 |
|  | Vertical | Large | Right | 10 |
|  | Vertical | Small | Left | 0 |
|  | Vertical | Small | Central | 4 |
|  | Vertical | Small | Right | 8 |
| Dyatel | Vertical | Large | Left | 1 |
|  | Vertical | Large | Central | 9 |
|  | Vertical | Large | Right | 9 |
|  | Vertical | Small | Left | 2 |
|  | Vertical | Small | Central | 8 |
|  | Vertical | Small | Right | 7 |
| Joe | Vertical | Large | Left | 0 |
|  | Vertical | Large | Central | 7 |
|  | Vertical | Large | Right | 11 |
|  | Vertical | Small | Left | 0 |
|  | Vertical | Small | Central | 4 |
|  | Vertical | Small | Right | 14 |
| Rodya | Horizontal | Large | Left | 11 |
|  | Horizontal | Large | Central | 12 |
|  | Horizontal | Large | Right | 10 |
|  | Horizontal | Small | Left | 1 |
|  | Horizontal | Small | Central | 2 |
|  | Horizontal | Small | Right | 0 |
| Dyatel | Horizontal | Large | Left | 0 |
|  | Horizontal | Large | Central | 12 |
|  | Horizontal | Large | Right | 2 |
|  | Horizontal | Small | Left | 0 |
|  | Horizontal | Small | Central | 22 |
|  | Horizontal | Small | Right | 0 |
| Joe | Horizontal | Large | Left | 8 |
|  | Horizontal | Large | Central | 12 |
|  | Horizontal | Large | Right | 9 |
|  | Horizontal | Small | Left | 0 |
|  | Horizontal | Small | Central | 5 |
|  | Horizontal | Small | Right | 2 |

# Table 2. The number of passes through the holes in group 2 in experiment 1

| Crow | Tipe of the hole | | | Number of passes |
| --- | --- | --- | --- | --- |
|  | Hole orientation | Hole size | Hole position |  |
| Glaz | Horizontal | Large | Left | 2 |
|  | Horizontal | Large | Central | 12 |
|  | Horizontal | Large | Right | 0 |
|  | Vertical | Large | Left | 6 |
|  | Vertical | Large | Central | 15 |
|  | Vertical | Large | Right | 10 |
|  | Horizontal | Small | Left | 2 |
|  | Horizontal | Small | Central | 20 |
|  | Horizontal | Small | Right | 0 |
|  | Vertical | Small | Left | 3 |
|  | Vertical | Small | Central | 1 |
|  | Vertical | Small | Right | 1 |
| Schnobel | Horizontal | Large | Left | 1 |
|  | Horizontal | Large | Central | 13 |
|  | Horizontal | Large | Right | 1 |
|  | Vertical | Large | Left | 6 |
|  | Vertical | Large | Central | 12 |
|  | Vertical | Large | Right | 16 |
|  | Horizontal | Small | Left | 0 |
|  | Horizontal | Small | Central | 20 |
|  | Horizontal | Small | Right | 1 |
|  | Vertical | Small | Left | 0 |
|  | Vertical | Small | Central | 1 |
|  | Vertical | Small | Right | 1 |
| Malyschka | Horizontal | Large | Left | 2 |
|  | Horizontal | Large | Central | 13 |
|  | Horizontal | Large | Right | 5 |
|  | Vertical | Large | Left | 0 |
|  | Vertical | Large | Central | 10 |
|  | Vertical | Large | Right | 14 |
|  | Horizontal | Small | Left | 1 |
|  | Horizontal | Small | Central | 11 |
|  | Horizontal | Small | Right | 4 |
|  | Vertical | Small | Left | 0 |
|  | Vertical | Small | Central | 6 |
|  | Vertical | Small | Right | 6 |

# Table 3. The number of first in trial attempts to pass through the holes in group 1 in experiment 2

| Crow | Tipe of the hole | | | Number of first in trial attempts to pass |
| --- | --- | --- | --- | --- |
|  | Hole passability | Hole position | Larger hole orientation |  |
| Rodya | Passable | Left | Horizontal | 2 |
|  | Passable | Central | Horizontal | 2 |
|  | Passable | Right | Horizontal | 2 |
|  | Passable | Left | Vertical | 2 |
|  | Passable | Central | Vertical | 2 |
|  | Passable | Right | Vertical | 2 |
|  | Impassable | Left | Horizontal | 0 |
|  | Impassable | Central | Horizontal | 0 |
|  | Impassable | Right | Horizontal | 0 |
|  | Impassable | Left | Vertical | 0 |
|  | Impassable | Central | Vertical | 0 |
|  | Impassable | Right | Vertical | 0 |
| Joe | Passable | Left | Horizontal | 2 |
|  | Passable | Central | Horizontal | 2 |
|  | Passable | Right | Horizontal | 2 |
|  | Passable | Left | Vertical | 2 |
|  | Passable | Central | Vertical | 2 |
|  | Passable | Right | Vertical | 2 |
|  | Impassable | Left | Horizontal | 0 |
|  | Impassable | Central | Horizontal | 0 |
|  | Impassable | Right | Horizontal | 0 |
|  | Impassable | Left | Vertical | 0 |
|  | Impassable | Central | Vertical | 0 |
|  | Impassable | Right | Vertical | 0 |
| Dyatel | Passable | Left | Horizontal | 2 |
|  | Passable | Central | Horizontal | 2 |
|  | Passable | Right | Horizontal | 2 |
|  | Passable | Left | Vertical | 2 |
|  | Passable | Central | Vertical | 2 |
|  | Passable | Right | Vertical | 1 |
|  | Impassable | Left | Horizontal | 0 |
|  | Impassable | Central | Horizontal | 0 |
|  | Impassable | Right | Horizontal | 0 |
|  | Impassable | Left | Vertical | 0 |
|  | Impassable | Central | Vertical | 1 |
|  | Impassable | Right | Vertical | 0 |

# Table 4. The number of first in trial attempts to pass through the holes in group 2 in experiment 2

| Crow | Tipe of the hole | | | Number of first in trial attempts to pass |
| --- | --- | --- | --- | --- |
|  | Hole passability | Hole position | Larger hole orientation |  |
| Glaz | Passable | Left | Horizontal | 2 |
|  | Passable | Central | Horizontal | 2 |
|  | Passable | Right | Horizontal | 2 |
|  | Passable | Left | Vertical | 2 |
|  | Passable | Central | Vertical | 1 |
|  | Passable | Right | Vertical | 2 |
|  | Impassable | Left | Horizontal | 0 |
|  | Impassable | Central | Horizontal | 0 |
|  | Impassable | Right | Horizontal | 0 |
|  | Impassable | Left | Vertical | 0 |
|  | Impassable | Central | Vertical | 1 |
|  | Impassable | Right | Vertical | 0 |
| Schnobel | Passable | Left | Horizontal | 2 |
|  | Passable | Central | Horizontal | 2 |
|  | Passable | Right | Horizontal | 2 |
|  | Passable | Left | Vertical | 2 |
|  | Passable | Central | Vertical | 2 |
|  | Passable | Right | Vertical | 1 |
|  | Impassable | Left | Horizontal | 0 |
|  | Impassable | Central | Horizontal | 1 |
|  | Impassable | Right | Horizontal | 0 |
|  | Impassable | Left | Vertical | 0 |
|  | Impassable | Central | Vertical | 0 |
|  | Impassable | Right | Vertical | 0 |
